# Determination of Tryptophan Metabolites in Serum and Cerebrospinal Fluid Samples Using Microextraction by Packed Sorbent, Silylation and GC–MS Detection

**DOI:** 10.3390/molecules25143258

**Published:** 2020-07-17

**Authors:** Alisa Pautova, Zoya Khesina, Maria Getsina, Pavel Sobolev, Alexander Revelsky, Natalia Beloborodova

**Affiliations:** 1 Laboratory of Human Metabolism in Critical States, Negovsky Research Institute of General Reanimatology, Federal Research and Clinical Center of Intensive Care Medicine and Rehabilitology, Petrovka str., 25-2, 107031 Moscow, Russia; mgetsina@mail.ru (M.G.); nvbeloborodova@yandex.ru (N.B.); 2 A.N. Frumkin Institute of Physical Chemistry and Electrochemistry, Russian Academy of Sciences, GSP-1, Leninsky Prospect, 31, 119071 Moscow, Russia; maldi-ms@yandex.ru; 3 Laboratory of Mass Spectrometry, Chemistry Department, Lomonosov Moscow State University, GSP-1, Leninskie gory, 1-3, 119991 Moscow, Russia; sobolevpd@mail.ru (P.S.); sorbent@yandex.ru (A.R.)

**Keywords:** tryptophan metabolites, indole-3-acetic acid, 5-hydroxyindole-3-acetic acid, indole-3-propionic acid, indole-3-lactic acid, microextraction by packed sorbent, silylation, gas chromatography–mass spectrometry

## Abstract

Indole-containing acids—tryptophan metabolites—found in serum and cerebrospinal fluid (CSF) samples of patients with diseases of the central nervous system (CNS) were determined with the use of microextraction by packed sorbent (MEPS) followed by silylation and gas chromatography–mass spectrometry (GC–MS) analysis. MEPS with the following silylation led to the reproducible formation of derivatives with an unsubstituted hydrogen ion in the indole ring, the chromatographic peaks of which are symmetric and can be used for GC–MS analysis without additional derivatization. The recoveries of analytes at the limit of quantitation (LOQ) levels were 40–80% for pooled CSF and 40–60% for serum. The limit of detection (LOD) and LOQ values were 0.2–0.4 and 0.4–0.5 µM, respectively, for both CSF and serum. The precision (the reproducibility, RSD) value of less than 20% and the accuracy (the relative error, RE) value of less than ±20% at the LOQ concentrations meet the Food and Drug Administration (FDA) recommendations. Linear correlations for all analytes were determined over a potentially clinically significant range of concentrations (0.4–10 µM for serum, *R*^2^ ≥ 0.9942, and 0.4–7 µM for CSF, *R*^2^ ≥ 0.9949). Moreover, MEPS significantly reduced the matrix effect of serum compared to liquid–liquid extraction (LLE), which was revealed in the example of reducing the amount of cholesterol and its relative compounds.

## 1. Introduction

The study of the metabolism of aromatic α-amino acids in the human body has been of scientific interest since the middle of the last century. Transformations of tyrosine, phenylalanine and tryptophan have both normal pathways in a healthy body and alternative pathways associated with various pathologies [1,2,3].

An important factor of tyrosine and phenylalanine metabolism is their microbial biodegradation to certain aromatic metabolites in the human body under the influence of normal or disturbed gut microbiota [4]. Some aromatic acids (benzoic, phenylacetic, phenylpropionic, phenyllactic, and *p*-hydroxyphenyllactic acids) are ubiquitous among healthy people and are detected in their serum in low concentrations. The concentrations of these compounds, as well as the appearance of other metabolites (homovanillic, *p*-hydroxyphenylacetic, and *p*-hydroxybenzoic acids), became diagnostically important, indicating the possible development of severe infectious complications [5,6]. The determination of aromatic metabolites in critically ill patients with sepsis, septic shock, and multiple organ failure, was carried out in blood serum. Several protocols for the determination of these compounds were developed. They include liquid–liquid extraction (LLE), microextraction by packed sorbent (MEPS) with C18 and hypercrosslinked polystyrene as sorbents with the following derivatization and gas chromatography – mass spectrometry (GC–MS) analysis [7,8,9].

Tryptophan metabolism occurs in two main ways—the indole (“I”) and kynurenine (“K”) pathways [10]. The indole metabolism (“I”) is conventionally divided into two main pathways— the serotonin or endogenous (“I-S”) pathway and the microbial (“I-M”) pathway. 5-Hydroxyindole-3-acetic acid (5HIAA) appears as a result of the serotonin metabolism (“I-S”) [11]. The microbial pathway (“I-M”) leads to the formation of metabolites containing an indole ring, for example, tryptamine (“I-M-T”), which, in particular, further forms indole-3-acetic (3IAA) and indole-3-propanoic (3IPA) acids [12]. Another way that tryptophan biodegradation can occur involves the formation of indole-3-pyruvic acid (“I-M-P”), which in turn gives 3IAA, indole-3-carboxylic (3ICA) and indole-3-lactic (3ILA) acids [13]. 3ILA is also known to be a microbial metabolite [14]. Thus, the detection and monitoring of the content of indolic acids, including those of microbial origin, is an interesting and promising issue [3]. Thus, the results of our studies, devoted to the role of the aromatic acids in the disorders of tyrosine and phenylalanine metabolism, will be equally helpful in understanding the tryptophan metabolism.

All mentioned metabolites (5HIAA, 3IAA, 3ILA, 3ICA, and 3IPA) have been detected at the nM-µM level both in healthy people and in patients with different pathologies, and this information is accumulated in the Human Metabolome Database (HMDB) [15]. 5HIAA and 3IAA are the most studied metabolites. They were detected in all common biological samples; 3ILA was detected in blood, feces, and urine; 3IPA was detected in blood, feces, and saliva; 3ICA was detected in feces.

Indolic acids can be detected in serum [16,17,18] or plasma [13,19] and cerebrospinal fluid (CSF) [20,21] samples using GC–MS [16,17,18,19] and high-performance liquid chromatography-mass spectrometry (HPLC–MS) [16] methods. LLE [13,17,18,19] and solid-phase extraction [22] are applied as sample preparation techniques. However, MEPS, which is one of the modern sample preparation techniques has not been described for indolic compound extraction. Thus, indolic acids are usually analyzed using the same methods as those for aromatic acids. The main difference of these two classes of compounds is the presence of the indole ring in indolic acids. Hydrogen ions in amino groups are more difficult to derivatize using conventional silylation reagents than in –OH and –COOH groups and significant excess of derivatizing reagent or additional derivatization is often required [23]. We can assume that the conditions that have been previously developed for the extraction of aromatic acids are suitable for indolic acids, taking into account the possible features of their derivatization. The aim of our study was the search for the sample preparation and silylation conditions for the determination of indolic compounds in serum and CSF samples using GC–MS analysis.

## 2. Results and Discussion

### 2.1. Mass Spectrometric Characteristics of the Silyl Derivatives of Indolic Acids

At the beginning of the study, the acetone aliquots of indolic acids were derivatized using *N*,*O*-bis(trimethylsilyl)trifluoroacetamide (BSTFA). The GC–MS analysis of the derivatives revealed that two trimethylsilyl (TMS) derivatives were formed for each indolic acid (Table 1). There were TMS forms corresponded to the completely substituted derivatives where all labile hydrogen ions were substituted by the TMS group (for 3IAA, 3ICA, and 3IPA—di-TMS derivatives; for 3ILA and 5HIAA—tri-TMS derivatives) and TMS forms with an unsubstituted hydrogen ion in the indole ring (for 3IAA, 3ICA, and 3IPA–mono-TMS derivatives; for 3ILA and 5HIAA—di-TMS derivatives).

**Figure 1:**
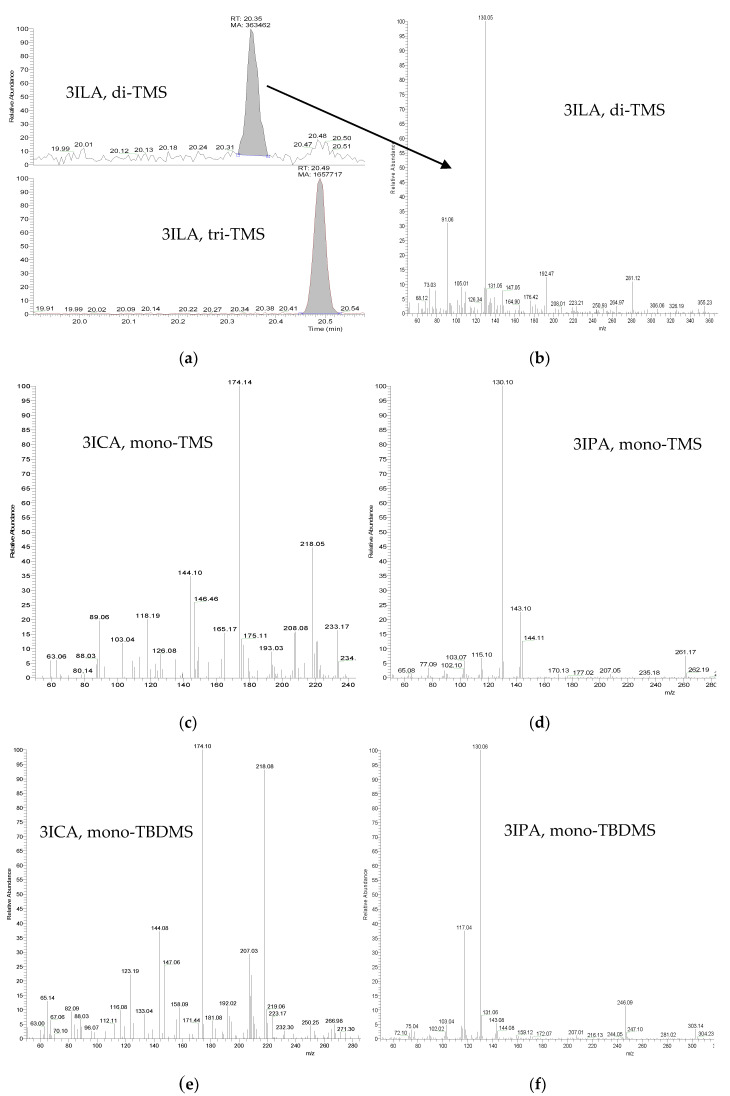
Fragments of mass chromatograms obtained using *m*/*z* values (Table 1) of di- and tri- trimethylsilyl (TMS) derivatives of indole-3-lactic acid (3ILA) (**a**), the mass spectra of the di-TMS derivative of 3ILA (**b**), the mono-TMS derivatives of indole-3-carboxylic acid (3ICA) (**c**) and indole-3-propionic acid (3IPA) (**d**), and the mono- *tert*-butyldimethylsilyl (TBDMS) derivatives of 3ICA (**e**) and 3IPA (**f**).

Figure 1a demonstrates the mass chromatogram fragments of TMS derivatives of 3ILA. The peak retention time (*Rt*), 20.49 min, corresponds to the tri-TMS derivative (completely substituted form). The peak with *Rt =* 20.35 min corresponds to the di-TMS derivative (incompletely substituted form) the mass spectrum of which is absent from the National Institute of Standards and Technology (NIST) mass spectral library (Figure 1b). The mass spectra of mono-TMS derivatives of 3ICA (Figure 1c) and 3IPA (Figure 1d) are also absent from the NIST mass spectral library. Other TMS derivatives of the analytes are present in NIST mass spectral library.

The mass spectrometric peak with *m*/*z* = 130 in the mass spectra of di-TMS derivative of 3ILA and mono-TMS derivative of 3IPA corresponds to the [M-CH(OSi(CH_3_)_3_)-COOSi(CH_3_)_3_]^+^ and [M-CH_2_COOSi(CH_3_)_3_]^+^ ions, respectively. Peaks with *m*/*z* = 261, *m*/*z* = 143 and *m*/*z* = 144 in the mass spectrum of the mono-TMS derivative of 3IPA correspond to the molecular radical cation, [M-HCOOSi(CH_3_)_3_]^+^ radical cation and the [M-COOSi(CH_3_)_3_]^+^ ion, respectively. Peaks with *m*/*z* = 233 and *m*/*z* = 218 in mass spectrum of mono-TMS derivative of 3ICA correspond to the molecular radical cation and [M-CH_3_]^+^ ion, respectively (Table 1). The peak with *m*/*z* = 174 corresponds to the [C_10_H_12_NSi]^+^ ion, which, presumably, may form as a result of the skeletal rearrangement process in the indole ring with the elimination of neutral particle.

Fragmentations of both completely and incompletely substituted derivatives proceed according to similar schemes (Table 1). For example, for most analytes, molecular radical cation was usually detected; for both forms of 3IAA, [M-COOSi(CH_3_)_3_]^+^ ions are characteristic; for 3ICA—[M-CH_3_]^+^ ions, for 3IPA—[M-CH_2_COOSi(CH_3_)_3_]^+^ ions, for 3ILA—[M-CH(OSi(CH_3_)_3_)-COOSi(CH_3_)_3_]^+^. It confirms that the recorded mass spectra absent from the NIST mass spectral library correspond to the incompletely substituted TMS derivatives of 3ICA, 3IPA and 3ILA. The peak forms of the incompletely substituted derivatives of indolic acids are symmetric and can be used in quantitative analysis.

The selection of derivatization conditions allowed us to obtain only incompletely substituted derivatives of indolic acids using BSTFA (40 µL, 90 °C, 30 min). The results were reproducible and the relative signals (peak areas of the analytes divided by the peak area of internal standard) of the TMS derivatives of indolic acids were the following: 3IAA 0.7 ± 0.2, 3ICA 0.35 ± 0.03, 3IPA 1.3 ± 0.1, 3ILA 0.8 ± 0.2, 5HIAA 0.5 ± 0.1. Subsequently they were used to calculate the recoveries of indolic acids from model solutions and biological samples in Section 2.3.

Derivatization using MTBSTFA with the formation of *tert*-butyldimethylsilyl (TBDMS) derivatives as an alternative silylating reagent was studied. The *Rt* values of obtained TBDMS derivatives were 2–4 min higher than for TMS derivatives (Table 1). All TBDMS derivatives of indolic acids were in an incompletely substituted form with an unsubstituted hydrogen ion in the indole ring. The mass spectra of the TBDMS derivatives of 3ICA and 3IPA are absent from the NIST mass spectral library (Figure 1e,f), but their fragmentation proceeds similarly to other TBDMS derivatives of indolic acids (Table 1). The mass spectrometric peak with *m*/*z* = 218 in the mass spectrum of the mono-TBDMS derivative of 3ICA and the peak with *m*/*z* = 249 in the mass spectrum of di-TBDMS derivative of 3IPA correspond to [M-C(CH_3_)_3_]^+^ ions, which are also formed during fragmentation of the TBDMS derivatives of 3IAA, 3ILA and 5HIAA. The peak with *m*/*z* = 130 in mass spectrum of 3IPA is also characteristic for 3IAA and 3ILA mass spectra; the peak with *m*/*z* = 117 corresponds to indole [C_8_H_7_N]^+^ radical cation. Thus, the recorded mass spectra that are not present in the NIST mass spectral library correspond to incompletely substituted TBDMS derivatives of 3ICA and 3IPA. The peak forms of the incompletely substituted TBDMS derivatives of indolic acids are symmetric and can be used for quantitative analysis as the results were reproducible. Therelative signals of TBDMS derivatives of indolic acids were the following: 3IAA 0.3 ± 0.1, 3ICA 0.012 ± 0.001, 3IPA 0.6 ± 0.2, 3ILA 0.90 ± 0.04, 5HIAA 0.17 ± 0.06. Subsequently, they were used to calculate the recoveries of indolic acids from model solutions in Section 2.3.

### 2.2. Retrospective Analysis of Indolic Acids in Biological Samples of Critically Ill Patients

We have developed the conditions based on LLE with diethyl ether, derivatization with BSTFA and GC–MS analysis for the determination of aromatic acids in serum samples of both healthy people and critically ill patients [7]. While investigating the profile of aromatic acids in serum samples of different groups of critically ill patients, we have accumulated a large amount of data. Since all chromatograms had been recorded in the full spectrum scan mode, we performed a qualitative retrospective analysis of indolic acid derivatives using *m*/*z* and *Rt* values, as described in Section 2.1.

The search for the chromatographic peaks of target compounds was made on the mass chromatograms of serum samples (*n* = 288) of critically ill patients with stroke, myocardial infarction, postoperative surgical complications, and sepsis. The derivatives of 3IAA, 3IPA, and 3ICA were found; the frequency of appearance was 91, 9, and 6%, respectively. Notably, 3ICA was not detected in serum samples according to HMDB. 3IPA was detected in di-TMS form (*n* = 25) and in mono-TMS form (*n* = 2). 3ICA was detected in di-TMS form (*n* = 16) and in mono-TMS form (*n* = 1).

3IAA (Table 2) was found in mono- (1) or di-TMS (2) forms, or in both forms (1 + 2). It was not possible to determine the regularity of appearance of 3IAA derivatives in different sets of samples; various ratios were observed between the occurrences of mono- or di-TMS derivatives, as well as between the ratio of their peak areas (*A*_1_ ˃ *A*_2_ or *A*_1_ ˂ *A*_2_).

The retrospective analysis of the chromatographic peaks of target compounds was also made on the mass chromatograms of the CSF samples (*n* = 138) of neurosurgical patients with various intracranial diseases or injuries. The derivatives of 3IAA, 3ILA, and 5HIAA were found; the frequency of appearance was 26, 2, and 0.7%, respectively. Notably, 3ILA was not detected in CSF samples according to HMDB. 3IAA was detected in di-TMS form (*n* = 36) and in mono-TMS form (*n* = 2). 3ILA was detected in tri-TMS form (*n* = 3); 5HIAA was detected in di-TMS form (*n* = 1).

To quantify the target components using calibration curves, it is important that the analytes are present in the chromatogram as a single derivative, and the formation of other forms is minimized. Thus, a quantitative analysis of 3IAA and other indolic acids in biological samples (especially in serum samples) using the conditions of LLE developed for aromatic acids is inappropriate.

### 2.3. Analytical Characteristics of MEPS for Indolic Acid Extraction

MEPS was chosen as an alternative sample preparation method to LLE. The conditions for the extraction of the indolic acids from serum and CSF samples were based on our previous study [8] and they were significantly modified. The main modification is the reduced to 40 µL volume of the biological sample, which is important for the analysis of CSF samples. To keep the sensitivity of the modified conditions, the number of sample loading cycles was increased, and the elution speed was reduced. The developed conditions are described in Section 3.2 and Section 3.3.

Preliminary experiments with model solutions showed that the application of MEPS with C18 sorbent and derivatization with BSTFA allows for detecting only incompletely substituted forms of indolic acids. Recoveries of the indolic acids from model solutions (concentration of addition was 100 µg/L/0.4–0.5 µM) using MEPS with BSTFA and MTBSTFA were comparable: 50–80% for BSTFA and 40–80% for MTBSTFA (Table 3). BSTFA was chosen for the further analyses for the following reasons: the recovery of 3IPA was higher for BSTFA; the reproducibility (RSD) of the recoveries of the indolic acids for BSTFA was better (10–23%) than for MTBSTFA (18–36%); *Rt* values for TMS derivatives were less than for TBDMS ones.

The recoveries from pooled CSF samples (40–80%) were comparable to those obtained from model solutions (50–80%) and higher than those from serum (40–60%). Recoveries at higher concentrations are also demonstrated in Table 3 and do not statistically differ from those for the limit of quantitation (LOQ) level.

**Figure 2:**
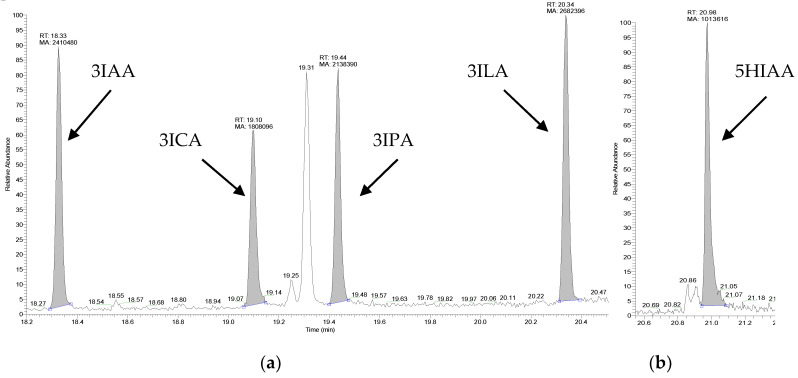

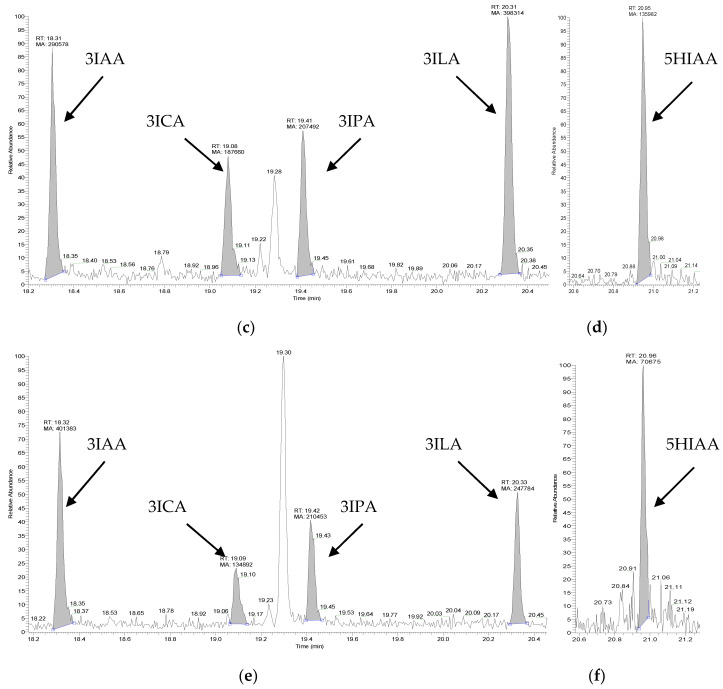
Mass chromatograms of a model solution (**a**,**b**), pooled cerebrospinal (CSF) sample (**c**,**d**) and serum sample of a healthy donor (**e**,**f**) after microextraction by packed sorbent (MEPS) with the addition of the indolic acids (concentration of each acid 500 µg/L / 2.0-2.5 µM) obtained using *m*/*z* values (130, 143, 174, 218, 247) of the trimethylsilyl (TMS) derivatives of indole-3-acetic acid (3IAA), indole-3-carboxylic acid (3ICA), indole-3-propionic acid (3IPA), indole-3-lactic acid (3ILA) (**a**,**c**,**e**) and *m*/*z* values (218, 335) of the TMS derivatives of 5-hydroxyindole-3-acetic acid (5HIAA) (**b**,**d**,**f**).

Linear correlations (Table 4) were determined over the 0.4–10 µM range of concentrations in model solutions (*R*^2^ ≥ 0.9960) and serum samples (*R*^2^ ≥ 0.9942) and over the 0.4–7 µM range of concentrations for pooled CSF samples (*R*^2^ ≥ 0.9949). The LOD values were 0.1–0.2 µM for model solutions, and 0.2–0.4 µM for pooled CSF and serum. The LOQ values were 0.4–0.5 µM for all matrices. The reproducibility (RSD) and relative error (RE) values for the LOQ concentration were less than 20% and ±20%, respectively (Table 4). The RSD and RE values at higher concentration levels also meet the necessary requirements, as described in Section 3.4. The obtained results can be illustrated by the mass chromatograms of a model solution (Figure 2a,b), pooled CSF (Figure 2c,d) and serum sample (Figure 2e,f) with the addition of the indolic acids. Thus, the developed conditions of MEPS can be used for the quantitative analysis of target compounds in CSF and serum samples.

### 2.4. Application of MEPS for Indolic Acid Extraction from Biological Samples of Critically Ill Patients

MEPS was used for the extraction of indolic compounds from serum (*n =* 3) and CSF (*n =* 3) samples of different patients with CNS diseases like cerebral infraction, meningitis and transient ischemic attack. Mono-TMS derivative of 3IAA was detected in all samples (*n* = 3, *p* = 0.95):

- CSF samples: #1–(0.42 ± 0.08) µM; #2–(0.6 ± 0.1) µM; #3–(0.43 ± 0.03) µM.
- Serum samples: #A–(0.47 ± 0.09) µM; #B–(0.43 ± 0.06) µM; #C–(0.6 ± 0.1) µM.

Other acids were not detected in the analyzed samples. We can explain the absence of 3IPA and 3ICA in serum samples, 3ILA and 5HIAA in CSF samples by their low frequency of appearance (9, 6, 2, and 0.7%, respectively) described in Section 2.2, and more thorough and extended research is required.

### 2.5. MEPS for Reducing the Matrix Effect

**Figure 3:**
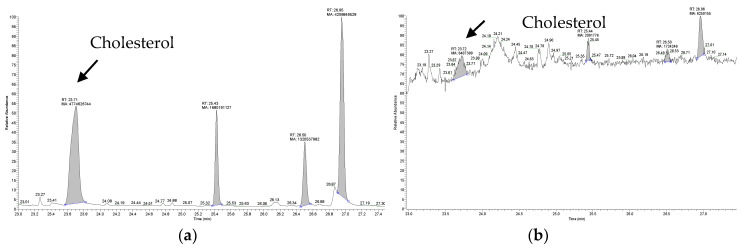
Fragments of chromatograms recorded in full spectrum scan mode obtained after the liquid-liquid extraction (LLE) (**a**) and the microextraction by packed sorbent (MEPS) (**b**) of the serum samples of a critically ill patient which indicate the content of interfering matrix components.

The *Rt* values of the indolic derivatives are close to those of the compounds with higher boiling points. These matrix compounds are usually coextracted with target compounds when using LLE, leading to the contamination of chromatography and mass spectrometry systems, long GC analysis times, and high final temperatures [7]. The utilization of MEPS led to the interesting results in solving this problem. Fragments of the chromatograms recorded in full spectrum scan mode obtained after LLE (Figure 3a) and MEPS (Figure 3b) of the serum samples of a critically ill patient indicated several peaks: *Rt* = 23.71 min corresponds to the TMS derivative of cholesterol; *Rt* = 25.43, 26.50 and 26.96 min correspond to the derivatives of related sterol compounds. MEPS with C18 sorbent demonstrated perfect results in reducing the content of these interfering matrix components even in the serum sample of a critically ill patient.

## 3. Materials and Methods

### 3.1. Chemicals and Reagents

Indole-3-carboxylic acid (3ICA, 99%), indole-3-acetic acid (3IAA, 98%), indole-3-propionic acid (3IPA, 99%), indole-3-lactic acid (3ILA, 99%), 5-hydroxyindole-3-acetic acid (5HIAA, ≥98%), 2,3,4,5,6-D_5_-benzoic acid (internal standard, D_5_-BA, ≥99 atom % D, ≥99%), *N*,*O*-bis(trimethylsilyl)trifluoroacetamide (BSTFA, contains 1% trimethylchlorosilane, 99% BSTFA), *N*-(*tert*-butyldimethylsilyl)-*N*-methyltrifluoroacetamide (MTBSTFA, >97%), formic acid (≥95%), hexane (≥97.0%), methanol (≥99.9%) were purchased from Sigma-Aldrich (Darmstadt, Germany); sulfuric acid, acetone, diethyl ether were Laboratory Reagent grade and obtained from Khimreactiv (Staryy Oskol, Russia).

### 3.2. Analysis of Model and Biological Samples

A mixture of aqueous stock solutions of each indolic acid (0.8 mg/L) and internal standard (1.0 mg/L) were prepared. Then, the aliquot of this mixture was added to the serum or pooled CSF solution to prepare solutions with concentrations of 100 (0.4–0.5 µM), 500 (2.0–2.5 µM), 1000 (4–5 µM) and 2000 (8–10 µM) µg/L to create respective calibration curves in the 0.4–10 µM range of concentrations for serum samples; 100 (0.4–0.5 µM), 500 (2.0–2.5 µM) and 1500 (6–7 µM) µg/L to create respective calibration curves in the 0.4–7 µM range of concentrations for pooled CSF samples.

Serum (*n* = 3), CSF (*n* = 3) samples of different patients with CNS diseases such as cerebral infraction, meningitis and transient ischemic attack and a healthy donor were collected in the Federal Research and Clinical Center of Intensive Care Medicine and Rehabilitology (Moscow, Russia). The approval of the local ethics committee was obtained (N 26/2019). Serum and CSF samples were stored at −30 °C for not more than 3 months and were defrosted at room temperature prior to use. Pooled CSF samples were used due to the absence of those of healthy donors and were prepared by mixing CSF samples from several patients with unconfirmed bacterial or viral infection of the CNS.

**Scheme 1:**
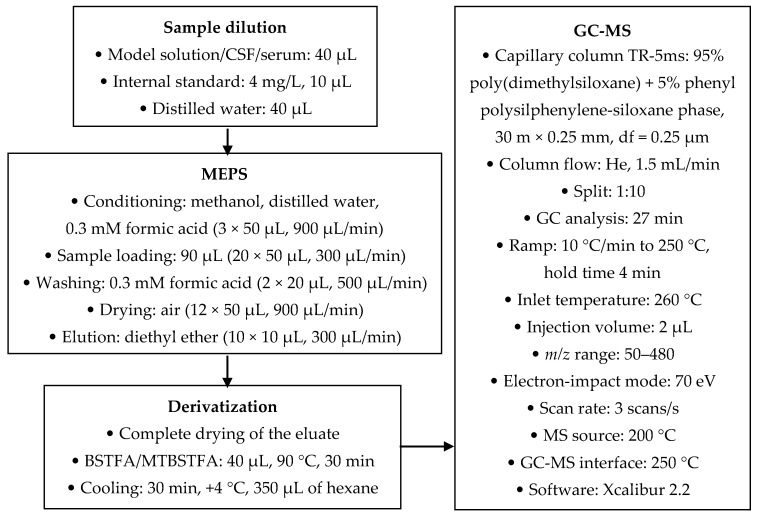
Conditions for the analysis of indolic acids.

The equipment for the analysis was the following: Trace GC 1310 gas chromatograph with an ISQ LT mass spectrometer and AI 1310 autosampler (Thermo Scientific, Thermo Electron Corporation, Waltham, MA, USA). 2,3,4,5,6-D_5_-benzoic acid was used as an internal standard for indolic acids. Briefly, the conditions for the analysis of the indolic acids are described in Scheme 1.

### 3.3. MEPS Procedure

MEPS was performed using a 50 µL volume syringe coupled with barrel insert and needle assemblies packed with ~4 mg of C18 (SGE Analytical, Melbourne, Australia). Prior to each sample preparation, the C18 sorbent was activated by consistent conditioning with methanol, distilled water and 0.3 mM formic acid. A total of 50 µL of sample was passed through the C18 sorbent 20 times. Subsequently, the solid phase was washed with 20 µL of 0.3 mM formic acid to remove interferences. The elution of analytes using diethyl ether was conducted after the sorbent drying, which was achieved by the passing of the air through the sorbent for 12 times. The analytes were derivatized using BSTFA after the complete drying of the organic eluate.

### 3.4. Method Validation

The recoveries, linearity, LOD, LOQ, accuracy, and precision were evaluated according to the guidelines of the FDA (endogenous analysis section) [24].

#### 3.4.1. Recovery

The recoveries of indolic acids were calculated at three different concentration levels (100, 500, 1500 µg/L for pooled CSF and 100, 500, 2000 µg/L for serum samples) as follows:

*Recovery* (*%*) = (*A*/*A_St_*)/(*A*/*A_St_*)*_100%_* × 100,

*A*—peak area of the TMS derivative of the indolic acid;

*A_St_*—peak area of the TMS derivative of the internal standard;

(*A/A_St_*)—relative signal obtained after sample preparation using MEPS;

(*A/A_St_*)*_100%_*—relative signal obtained from organic solution without any kind of sample preparation (except derivatization).

#### 3.4.2. Calibration Curve

For the determination of linearity, the calibration curves for all analytes were revealed using the spiked serum and pooled CSF samples described in Section 3.2, which were treated by MEPS procedure. Calibration curves were obtained by plotting the ratio of the analyte peak area/internal standard peak area as a function of the corresponding nominal concentrations (three replicates for each concentration levels) and calculated using the least squares method.

#### 3.4.3. LOD and LOQ

LODs (µM) were calculated according to the following formula:

*LOD* (*µM*) = 3 × *SD*/*b*,

*SD*—the standard deviation;

*b*—the slope.

LOQs (µM) were defined as the lowest point of the calibration curve (100 µg/L / 0.4–0.5 µM).

#### 3.4.4. Accuracy and Precision

The precision was defined as RSD (%). The accuracy was defined as RE (%):

*RE* (%) = [((*measured concentration* − *concentration in a sample without additives*) − *nominal concentration*)/*nominal concentration*] × 100%.

The values of RSD not deviating more than 20% and RE not deviating more than ±20% from the nominal concentration at the LOQ were considered acceptable as well as RSD not deviating 15% and RE not deviating ±15% from the nominal concentration for standards other than LOQ.

#### 3.4.5. Selectivity

Selectivity for all analytes and internal standards was also evaluated for CSF by three parallel analysis of pooled CSF samples from three different patients and for serum by three parallel analysis of a healthy donor serum. After this, serum and pooled CSF samples were used in the preparation of calibration standards and were subsequently subjected to MEPS sample preparation and GC–MS analysis. Response of the interfering compounds did not exceed 20% of the LOQ values.

#### 3.4.6. Stability

The instability of aqueous solutions of indolic acids was observed and fresh stock solutions were prepared on a daily basis.

#### 3.4.7. Carryover Effects

The MEPS carryover effect was evaluated as follows: needle, which had been used for all previous extraction procedures with calibration standards, was used for extraction of pooled CSF or serum sample without the addition of indolic acids; the extract was derivatized and introduced into the chromatographic system. We did not observe an increase in the areas of chromatographic peaks during the elution of the target analytes, so the carryover effect of MEPS was considered to be insignificant.

The blank sample of the eluting solvent was introduced into the chromatographic system immediately after the analysis of all calibration standards to evaluate the carryover effect of the chromatographic system, which did not exceed 0.1% of the areas of chromatographic peaks of indolic acids obtained during the previous analysis.

### 3.5. Statistical Analysis

The statistical processing of experimental data was performed based on the results of three parallel experiments and represented the mean with a confidence interval (*n* = 3, *p* = 0.95). All data were analyzed using Microsoft Excel 2016.

## 4. Conclusions

Tryptophan metabolites were determined in serum and CSF samples of patients with CNS diseases using MEPS with the following derivatization and GC–MS analysis. These metabolites are indole-containing acids—3IAA, 5HIAA, 3IPA, 3ILA, and 3ICA; some of them are known to be of microbial origin. Silylation with BSTFA and MTBSTFA led to the reproducible formation of derivatives with an unsubstituted hydrogen ion in the indole ring. The mass spectra of some derivatives are described in detail because they are absent from the NIST mass spectral library. By analogy with the fragmentation of fully derivatized forms of indolic acids, we proposed a possible fragmentation for incompletely derivatized forms and showed that the fragmentation of both forms occurs according to similar schemes. The chromatographic peaks of all incompletely substituted derivatives are symmetric and can be used for the GC–MS analysis without additional derivatization.

The qualitative retrospective analysis of tryptophan metabolites on the chromatograms of serum (*n* = 288) and CSF (*n* = 138) samples of different groups of patients using LLE revealed interesting facts. The derivatives of 3IAA were found both in serum and CSF (the frequency of appearance was 91 and 26%, respectively); 3IPA and 3ICA were found in serum (the frequency of appearance was 9 and 6%, respectively); 3ILA and 5HIAA were found in CSF (the frequency of appearance was 2 and 0.7%, respectively). The facts that 3ICA was found in serum samples and 3ILA was found in CSF samples are not described in HMDB. However, quantitative analysis of indolic acids using LLE is inappropriate because of the irreproducible formation of different silyl derivatives of analytes.

The advantages of MEPS were shown in comparison with the LLE. An aliquot of only 40 µL of biological sample can be analyzed using MEPS instead of 200 µL for LLE. The utilization of MEPS, in contrast to LLE, makes it possible to carry out a quantitative analysis of indolic acids, since it allows for the reproducible formation of only one of the derivatized forms (incompletely substituted ones). Some analytical characteristics of the methodology were calculated, and their values met the requirements of the FDA recommendations. The applicability of the developed conditions was demonstrated by analyzing real samples, where 3IAA was reproducibly determined in the CSF and serum samples of patients with CNS diseases at the 0.4–0.6 µM level. Other acids were not detected at this stage of research and the analysis of biological samples of the extended group of patients is required. A comparison of the chromatograms of the same serum sample obtained after MEPS and LLE demonstrated that the utilization of MEPS significantly reduced the amount of sterol matrix components of serum compared to LLE. All of the mentioned advantages of MEPS with the C18 sorbent suggests that this approach can be an excellent alternative to traditional sample preparation techniques for the analysis of small target analytes in complex matrices such as blood serum and CSF.

## Figures and Tables

**Table 1:** Retention times (*Rt*), *m*/*z* values and possible structure of characteristic ions of indolic acid derivatives (M^+^ - molecular radical cation).

Indolic Acid, Derivative, Structure	*Rt*, min	M, Da	*m*/*z*	Possible Structure of Characteristic Ions
Trimethylsilyl (TMS) Derivatives				
Indole-3-acetic acid (3IAA), mono- (CH_3_)_3_SiOOCCH_2_-C_8_H_6_N	18.26	247	130 247	[M-COOSi(CH_3_)_3_]^+^ M^+^
3IAA, di- (CH_3_)_3_SiOOCCH_2_-C_8_H_5_NSi(CH_3_)_3_	18.60	319	202 319	[M-COOSi(CH_3_)_3_]^+^ M^+^
Indole-3-carboxylic acid (3ICA), mono-^1^ (CH_3_)_3_SiOOC-C_8_H_6_N	19.12	233	218 233	[M-CH^3^]^+^ M^+^
3ICA, di- (CH_3_)_3_SiOOC-C_8_H_5_NSi(CH_3_)_3_	19.21	305	290 305	[M-CH^3^]^+^ M^+^
Indole-3-propionic acid (3IPA), mono-^1^ (CH_3_)_3_SiOOC(CH_2_)_2_-C_8_H_6_N	19.44	261	130 261	[M-CH_2_COOSi(CH_3_)_3_]^+^ M^+^
3IPA, di- (CH_3_)_3_SiOOC(CH_2_)_2_-C_8_H_5_NSi(CH_3_)_3_	19.77	333	202 333	[M-CH_2_COOSi(CH_3_)_3_]^+^ M^+^
Indole-3-lactic acid (3ILA), di-^1^ NC_8_H_6_-CH_2_CH(OSi(CH_3_)_3_)COOSi(CH_3_)_3_	20.35	349	130	[M-CH(OSi(CH_3_)_3_)-COOSi(CH_3_)_3_]^+^
3ILA, tri- (CH_3_)_3_Si-NC_8_H_5_-CH_2_CH(OSi(CH_3_)_3_)COOSi(CH_3_)_3_	20.49	421	202 421	[M-CH(OSi(CH_3_)_3_)-COOSi(CH_3_)_3_]^+^ M^+^
5-Hydroxyindole-3-acetic acid (5HIAA), tri- (CH_3_)_3_SiOOCCH_2_-C_8_H_4_NSi(CH_3_)_3_-OSi(CH_3_)_3_	20.88	407	290 407	[M-OSi(CH_3_)_3_; -CH_3_]^+^ M^+^
5HIAA, di- (CH_3_)_3_SiOOCCH_2_-C_8_H_5_N-OSi(CH_3_)_3_	20.99	335	218 335	[M-COOSi(CH_3_)_3_]^+^ M^+^
*Tert*-butyldimethylsilyl (TBDMS) Derivatives				
3IAA, mono- (CH_3_)_3_C-(CH_3_)_2_SiOOCCH_2_-C_8_H_6_N	20.54	289	130 232 289	[M-COOSi(CH_3_)_3_-C(CH_3_)_3_]^+^ [M-C(CH_3_)_3_]^+^ M^+^
3ICA, mono-^1^ (CH_3_)_3_C-(CH_3_)_2_SiOOC-C_8_H_6_N	21.52	275	218	[M-C(CH_3_)_3_]^+^
3IPA, mono-^1^ (CH_3_)_3_C-(CH_3_)_2_SiOOC(CH_2_)_2_-C_8_H_6_N	21.63	303	130 246 303	[M-CH_2_COOSi(CH_3_)_3_-C(CH_3_)_3_]^+^ [M-C(CH_3_)_3_]^+^ M^+^
3ILA, di- NC_8_H_6_-CH_2_CH(OSi(CH_3_)_2_- C(CH_3_)_3_)COOSi(CH_3_)_2_-C(CH_3_)_3_	23.83	433	130 376	[M-CH(OSi(CH_3_)_3_-C(CH_3_)_3_)-COOSi(CH_3_)_3_-C(CH_3_)_3_]^+^ [M-C(CH_3_)_3_]^+^
5HIAA, di- (CH_3_)_3_C-(CH_3_)_2_SiO-NC_8_H_5_-CH_2_COOSi(CH_3_)_2_-C(CH_3_)_3_	24.38	419	362 419	[M-C(CH_3_)_3_]^+^M^+^

^1^ Mass spectrum is absent from the NIST mass spectral library.

**Table 2:** Frequency of appearance of indole-3-acetic acid (3IAA) trimethylsilyl (TMS) derivatives in serum samples of critically ill patients using liquid–liquid extraction (LLE) as a sample preparation technique (*A*–peak area).

Group of Patients	Number of Samples	Presence in Samples	mono-TMS (1)	di-TMS (2)	1 + 2	*A*_1_ ˃ *A*_2_	*A*_1_ ˂ *A*_2_
1	38	35	21	27	13	8	5
2	24	23	8	23	7	4	3
3	64	59	58	14	13	11	2
4	55	55	51	53	49	31	18
5	107	91	56	70	35	25	10
Σ	288	263	194	187	118	79	38

**Table 3:** Recoveries (%) of the silyl derivatives of indolic acids from model solution (derivatization with *N*,*O*-bis(trimethylsilyl)trifluoroacetamide (BSTFA) or *N*-(*tert*-butyldimethylsilyl)-*N*-methyltrifluoroacetamide (MTBSTFA)), pooled cerebrospinal fluid (CSF) samples and serum samples at three concentration levels using microextraction by packed sorbent (MEPS) as a sample preparation technique and BSTFA as a derivatizing reagent (*n* = 3, *p* = 0.95).

Indolic Acid Derivative	Model Solution		Pooled CSF			Blood Serum		
	BSTFA	MTBSTFA	100 ^a^	500 ^b^	1500 ^c^	100 ^a^	500 ^b^	2000 ^d^
Indole-3-acetic acid (3IAA), mono-	80 ± 20	80 ± 10	80 ± 20	80 ± 20	80 ± 10	60 ± 20	60 ± 10	60 ± 20
Indole-3-carboxylic acid (3ICA), mono-	70 ± 10	70 ± 20	60 ± 20	70 ± 20	60 ± 10	60 ± 10	70 ± 20	60 ± 10
Indole-3-propionic acid (3IPA), mono-	62 ± 6	40 ± 10	70 ± 20	70 ± 10	70 ± 10	48 ± 9	50 ± 10	50 ± 10
Indole-3-lactic acid (3ILA), di-	80 ± 20	70 ± 20	80 ± 20	70 ± 20	80 ± 20	50 ± 10	50 ± 10	60 ± 10
5-Hydroxyindole-3-acetic acid (5HIAA), di-	52 ± 8	40 ± 10	40 ± 10	50 ± 10	50 ± 10	40 ± 10	38 ± 6	40 ± 10

^a^ 100 µg/L corresponds to 0.4–0.5 µM; ^b^ 500 µg/L corresponds to 2.0–2.5 µM; ^c^ 1500 µg/L corresponds to 6–7 µM; ^d^2000 µg/L corresponds to 8–10 µM.

**Table 4:** Analytical characteristics of the determination of the indolic acids in model solutions, pooled cerebrospinal (CSF) and serum samples (*n* = 3, *p* = 0.95).

Indolic Acid	Matrix	Dynamic Concentration Range, µM	*R* ^2^	LOD, µM	LOQ, µM	^1^RSD, %	^1^RE, %
Indole-3-acetic acid (3IAA)	Model solution	0.4–9	0.9982	0.1	0.4	15	−9
	Pooled CSF	0.4–7	0.9951	0.2	0.4	16	−11
	Blood serum	0.4–9	0.9945	0.2	0.4	19	15
Indole-3-carboxylic acid (3ICA)	Model solution	0.5–10	0.9973	0.1	0.5	7	10
	Pooled CSF	0.5–7	0.9998	0.3	0.5	10	20
	Blood serum	0.5–10	0.9987	0.4	0.5	20	−17
Indole-3-propionic acid (3IPA)	Model solution	0.4–8	0.9989	0.1	0.4	6	13
	Pooled CSF	0.4–6	0.9949	0.4	0.4	18	16
	Blood serum	0.4–8	0.9942	0.4	0.4	16	12
Indole-3-lactic acid (3ILA)	Model solution	0.4–8	0.9960	0.2	0.4	17	19
	Pooled CSF	0.4–6	0.9963	0.2	0.4	18	−20
	Blood serum	0.4–8	0.9967	0.3	0.4	18	13
5-Hydroxyindole-3-acetic acid (5HIAA)	Model solution	0.4–8	0.9981	0.1	0.4	7	−11
	Pooled CSF	0.4–6	0.9991	0.3	0.4	19	19
	Blood serum	0.4–8	0.9991	0.4	0.4	20	17

^1^RSD (relative standard deviation) and RE (relative error) are given for LOQ (limit of quantitation) level.

## Abbreviations

BSTFA	*N*,*O*-bis(trimethylsilyl)trifluoroacetamide
CNS	central nervous system
CSF	cerebrospinal fluid
FDA	Food and Drug Administration
GC–MS	gas chromatography–mass spectrometry
5HIAA	5-hydroxyindole-3-acetic acid
HMDB	Human Metabolome Database
3IAA	indole-3-acetic acid
3ICA	indole-3-carboxylic acid
3ILA	indole-3-lactic acid
3IPA	indole-3-propionic acid
LLE	liquid-liquid extraction
LOD	limit of detection
LOQ	limit of quantitation
MEPS	microextraction by packed sorbent
MTBSTFA	*N*-(*tert*-butyldimethylsilyl)-*N*-methyltrifluoroacetamide
NIST	National Institute of Standards and Technology
RE	relative error
RSD	relative standard deviation
Rt	retention time
TBDMS	*tert*-butyldimethylsilyl
TMS	trimethylsilyl
